# Revisiting visceral leishmaniasis in immunocompromised patients: Ongoing gaps and advances in diagnostics, therapies, and preventive measures

**DOI:** 10.1016/j.crpvbd.2026.100377

**Published:** 2026-04-09

**Authors:** Soroush Partovi Moghaddam, Iraj Sharifi, Narges Lotfalizadeh, Shayan Amini, Ali Mobaraki, Bita Fazel, Mehdi Bamorovat, Ahmad Khosravi, Baharak Akhtardanesh, Mehdi Mohebali

**Affiliations:** a Department of Pathobiology, Faculty of Veterinary Specialized Science, Science, and Research Branch, Islamic Azad University, Tehran, Iran; b Leishmaniasis Research Center, Kerman University of Medical Sciences, Kerman, Iran; c Department of Clinical Sciences, Faculty of Veterinary Medicine, Shiraz University, Shiraz, Iran; d Shahid Beheshti Medical University, Tehran, Iran; e Faculty of Veterinary Medicine, University of Tehran, Tehran, Iran; f Department of Clinical Sciences, Faculty of Veterinary Medicine, Shahid Bahonar University of Kerman, Kerman, Iran; g Department of Medical Parasitology and Mycology, School of Public Health, Tehran University of Medical Sciences, Tehran, Iran; h Center for Research of Endemic Parasites of Iran (CREPI), Tehran University of Medical Sciences, Tehran, Iran

**Keywords:** Visceral leishmaniasis, Immunosuppression, HIV/AIDS, Organ transplantation, Diagnosis, Treatment, Preventive measures

## Abstract

The visceral leishmaniasis (VL) caused by *Leishmania infantum* and *Leishmania donovani* is regarded as one of the deadliest forms of parasitic diseases, claiming thousands of victims a year, and is particularly prevalent in tropical and subtropical climates. Even though a healthy immune system can often combat or control the infection, in immunocompromised individuals, the disease emerges chronically, aggressively, and often fatally. In recent years, VL has become a significant public health threat in vulnerable populations due to the increased prevalence of conditions such as HIV/AIDS, hematological malignancies such as lymphoma and leukemia, and the increased use of immunosuppressive drugs among organ transplant recipients. This review aimed to provide an overview of the challenges and limitations related to diagnosing, preventing, and treating VL in immunocompromised patients, as well as their clinical and epidemiological consequences. An analysis of recent clinical and epidemiological data has indicated that common diagnostic tests, especially serological tests such as rK39 and DAT, are insufficiently sensitive and specific for immunocompromised patients, and that molecular methods such as PCR and qPCR are necessary to accurately diagnose and treat early. In the context of treatment, liposomal amphotericin B is still regarded as a first-line drug in many regions of the world, but its effectiveness has been significantly limited due to toxicity, drug resistance, and frequent relapses in immunocompromised individuals. Moreover, prevention programmes for this group of patients remain ineffective due to the lack of definitive preventive drugs and effective vaccines, as well as weak population screening. According to recent immunological studies, deficiencies in cellular immune responses, particularly impaired T helper cell (CD4+) function, defective IFN-γ production, and compromised macrophage function in parasite clearance, play a significant role in the complex pathogenesis of VL in immunosuppressed patients. In patients infected with HIV, *Leishmania* infection synergistically increases viral loads, reduces antiretroviral therapy response, and increases mortality. In conclusion, the results of this study indicate that VL among immunocompromised patients is not only clinically and therapeutically more challenging but may also, epidemiologically, play a hidden role in the survival of the human reservoir and the spread of the disease.

## Highlights

• Immunocompromised patients experience severe atypical visceral leishmaniasis with high relapse.
• Diagnosis is challenging, requiring advanced molecular/serological tools beyond traditional methods.
• Therapeutic strategies must balance efficacy, toxicity, and relapse in this high-risk population.
• Preventive measures like screening and vector control are critical to reducing disease burden in endemic areas.

## Graphical abstract

**Figure:**
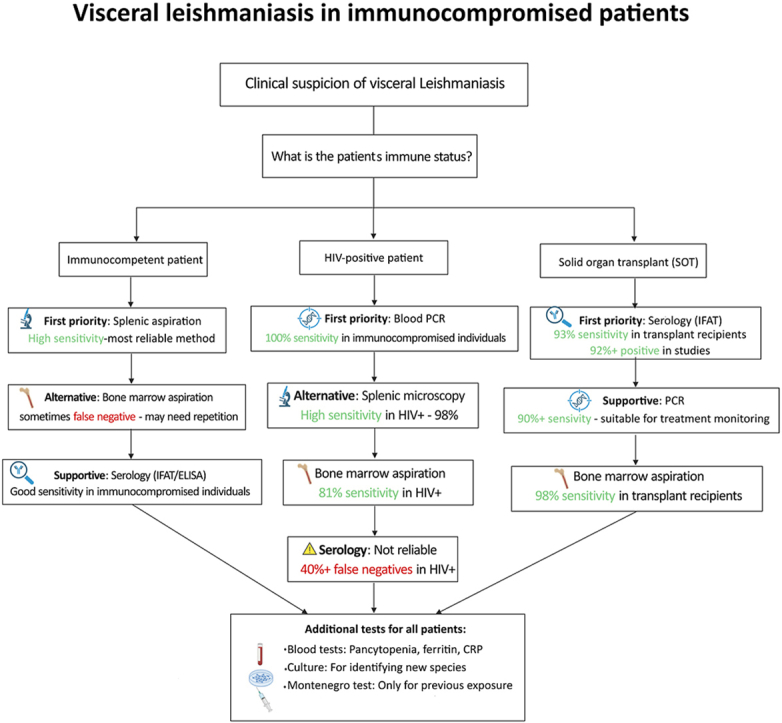

## 1 Introduction

Leishmaniasis is an internationally significant parasitic disease caused by 20 species of *Leishmania*, affecting humans and mammals in over 100 impoverished countries. Leishmaniasis, classified by the World Health Organization (WHO) as a neglected tropical disease (NTD), is a persistent public health challenge in many endemic areas worldwide and contributes significantly to mortality and disability, particularly among vulnerable populations (Bruschi and Gradoni, 2018; Grifferty et al., 2021). Leishmaniasis is one of the 20 NTDs, which collectively affect more than one billion people worldwide (Cosma et al., 2024). It presents primarily in three classical clinical forms: visceral (VL), cutaneous (CL), and mucocutaneous leishmaniasis (MCL) (Mohebali et al., 2023; Akhtardanesh et al., 2024; Sadr et al., 2025b). Leishmaniasis is generally transmitted by bites of sand flies of the genus *Phlebotomus* (in the Old World) and *Lutzomyia* (in the New World), by blood transfusions, or through contaminated syringes (Mansueto et al., 2014; Bhunia and Shit, 2020). Nevertheless, the spread of the prevalent species (*Leishmania tropica* and *Leishmania infantum*) within Europe may lead to the emergence or reappearance of leishmaniasis on the continent. The effects of climate change, population growth, migration, and the emergence of exotic species of *Leishmania* all contribute to the spread of this disease (Grifferty et al., 2021; Maia, 2024; Sadr et al., 2025a).

Visceral leishmaniasis, also known as Kala-Azar, caused by *Leishmania donovani* and *L. infantum/L. chagasi*, is the most severe clinical form of leishmaniasis and is a potentially fatal disease, leading to death in over 95% of untreated cases. It presents with intermittent fever, weight loss, anemia, and enlargement of the reticulo-endothelial organs, notably the spleen and liver (Jervis et al., 2017). The majority of cases are reported from Brazil, East Africa, and India. Globally, an estimated 50,000 to 90,000 new VL cases occur each year; nevertheless, nearly 25–45% are officially reported to the WHO. The disease poses significant risks for outbreaks and high mortality (Agura, 2024; Sadeghi et al., 2024). It is critical to note that clinical disease severity depends on the interaction between host immunity and parasite species and load (Costa et al., 2023a). The incidence of asymptomatic VL infections is higher in leishmaniasis-endemic countries than in cases with clinically observable VL. The proportion of people with asymptomatic VL who develop clinically overt VL is estimated at 10–20% (Banu et al., 2016). Visceral leishmaniasis is especially recognized as an opportunistic infection in immunosuppressed individuals (Ponte-Sucre et al., 2017). Malnutrition, HIV co-infections, organ transplants, and helminth infections are the leading immunosuppressive factors (Akuffo et al., 2018). Immunosuppression greatly increases the risk of developing overt clinical disease and alters the way the condition presents and responds to treatment. Since the early 1980s, the prevalence of immunosuppressed patients exposed to different infectious or neoplastic processes has increased every decade. This converged with the acquired immunodeficiency syndrome (AIDS) (Ramos et al., 2017). In recent years, transplants have expanded (bone marrow, liver, kidney, heart, etc.), and so has the development of novel immunosuppressive drugs, which are now widely used in these patients, as well as in patients with autoimmune diseases and neoplastic diseases. The cumulative number of patients with chronic immunosuppression increases their vulnerability to opportunistic infections. In this regard, the present review focuses on the diagnostic, therapeutic, and preventive challenges of visceral leishmaniasis in immunosuppressed patients.

T-helper cells (Th), especially Th-1, eliminate *Leishmania* spp. in healthy immunocompetent hosts. In immunosuppressed patients, T-cell responses are inadequate, and they are more likely to develop clinical disease, experience a more severe course, and have a higher relapse rate (Clemente et al., 2018a). Th-1 secretes cytokines Interleukin-2 (IL-2), Interferon-Gamma (INF-γ), and Tumor Necrosis Factor-α (TNF-α) that recruit macrophages and activate them to phagocytose *Leishmania* amastigotes (Toepp and Petersen, 2020).

There is a high rate of disease relapse in patients with impaired T-cell function or number (patients receiving corticosteroids or cytotoxic agents, transplant recipients, and patients with advanced HIV disease) (WHO, 2022). Proper treatment usually results in a long-term clinical cure; however, parasite eradication is unlikely in most patients. Although the disease can be cured clinically, it can recur several years afterwards. An effective way to detect parasites is through sensitive assays, such as polymerase chain reaction (PCR) (Mesa et al., 2020). Despite significant advances in the diagnosis and treatment of visceral leishmaniasis, the management of this disease in immunocompromised or immunosuppressed patients remains fraught with major challenges, including atypical clinical presentations, limited sensitivity of conventional diagnostic methods, and unpredictable therapeutic responses. Hence, this review focuses specifically on visceral leishmaniasis, as this form of the disease is a life-threatening risk and interacts strongly with immunosuppression.

## 2 Search strategies

This narrative review was conducted using a structured search strategy. Key concepts, including visceral leishmaniasis, immunocompromised populations, diagnostic methods, treatment strategies, and preventive measures, were identified. Related keywords and synonyms, including “*Leishmania donovani*” and “Kala-Azar” for visceral leishmaniasis; immunosuppression-related terms, including HIV/AIDS, malignancies, and immunosuppressive therapies; and terms related to diagnosis, treatment, and prevention, were defined and applied. A search was conducted in PubMed, Scopus, Web of Science, and the Cochrane Library for peer-reviewed articles published between January 2010 and January 2026, with a primary focus on immunocompromised populations. The initial search identified 1675 records, which were reduced to 1386 after duplicates were removed. After title and abstract screening, 670 articles were selected for full-text review, and 202 studies met the specified inclusion criteria and were included in the final analysis. Inclusion criteria included human studies relevant to the pathogenesis, clinical manifestations, diagnostic methods, therapeutic approaches, or outcomes of visceral leishmaniasis in immunocompromised patients. The included studies comprised original human studies and review articles related to the topic. Conference abstracts were not included in the final analysis. Animal or laboratory studies without direct clinical application, single case reports, articles lacking full text, and studies that only addressed immunocompetent populations were excluded.

## 3 Life cycle

**Fig. 1:**
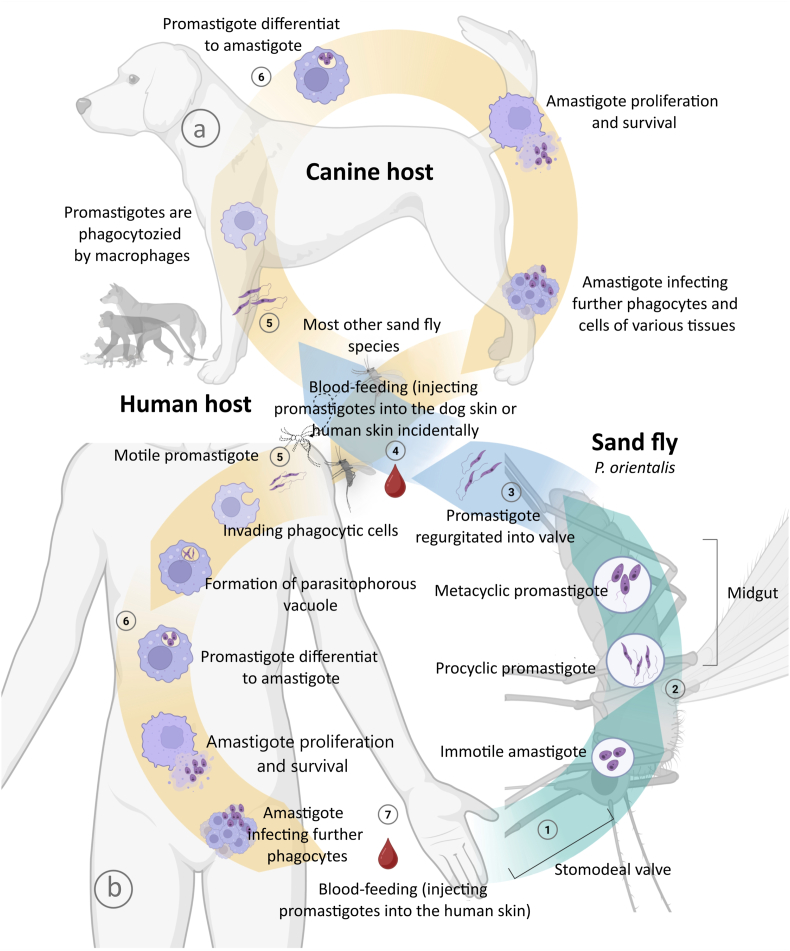
Life cycle of *Leishmania* parasites. *Sand fly vector stage*: The cycle begins when a female sand fly ingests immotile amastigotes during a blood meal from an infected host (**1**). Inside the sand fly midgut, amastigotes transform into procyclic promastigotes (**2**), which are motile and replicate by binary fission. As they migrate anteriorly, they differentiate into infective metacyclic promastigotes (**3**). During the next blood meal, promastigotes are regurgitated into the mammalian host’s skin through the stomodeal valve. *Mammalian host stage*: Once introduced into the host, motile promastigotes are phagocytized by macrophages **(4)**. Inside these immune cells, promastigotes become enclosed within parasitophorous vacuoles where they differentiate back into amastigote forms (**5**). Amastigotes multiply and survive in white blood cells, eventually spreading to infect additional macrophages and cells across various tissues, particularly within the reticuloendothelial system (**6**). *Zoonotic cycle* (*Leishmania infantum*): In the zoonotic transmission pattern (*Pathway****a***), dogs function as the primary reservoir host for *L. infantum. Phlebotomus* (Old World) or *Lutzomyia* (New World) sand flies maintain transmission between canine reservoirs. Humans serve as incidental hosts when bitten by infected sandflies (**5**-**6**-**7**, *Pathway****b***), with the parasite undergoing identical developmental stages within human tissues as it does in dogs. *Anthroponotic cycle* (*Leishmania donovani*): *L. donovani* follows an anthroponotic transmission system with humans as the principal reservoir (*Pathway****b***). Sand flies, specifically *Phlebotomus argentipes* and *Phlebotomus orientalis*, are directly capable of transferring the parasite causing this disease from one human host to another without any involvement of an animal reservoir as part of the transmission process. This human-to-sand fly-to-human cycle undergoes the same intracellular developmental stages but has significant epidemiological implications, as disease control depends entirely on reducing human infectious reservoirs through early detection and treatment rather than animal reservoir management. Created in https://BioRender.com.

*Leishmania infantum/L. chagasi* and *Leishmania donovani* share a digenetic life cycle involving sand fly vectors and mammalian hosts, but differ in transmission dynamics, i.e. zoonotic *versus* anthroponotic, reflecting distinct epidemiological patterns. *Leishmania infantum* (syn. *L. chagasi* in the Americas) is the etiological agent of zoonotic visceral leishmaniasis (ZVL), prevalent in the Mediterranean basin and Latin America (Scarpini et al., 2022). Its life cycle begins when a female phlebotomine sand fly ingests infected macrophages containing amastigotes during a blood meal. Within the sand fly midgut, amastigotes transform into motile promastigotes, multiply, and migrate to the proboscis. Upon subsequent feeding, the sand fly injects metacyclic promastigotes into a mammalian host (typically dogs), which serve as the primary reservoir. In the host, promastigotes are phagocytosed by macrophages, which then revert to amastigotes, proliferate intracellularly, and disseminate through the reticuloendothelial system (Fig. 1(a)). This zoonotic cycle is maintained between sand flies and reservoir mammals, infrequently transmitting to humans (Alizadeh et al., 2025). In regions where human ZVL is widespread, the deployment of control measures focused on dogs may be of dual relevance. These methods can protect dogs from the suffering caused by the illness while also decreasing the high risk of disease transmission to humans (Abbaszadeh Afshar et al., 2018; Mohebali et al., 2018; Yimam and Mohebali, 2020).

Conversely, *L*. *donovani* causes anthroponotic visceral leishmaniasis (AVL), notably in the Indian subcontinent, where humans are the sole significant reservoir (Fatollahzadeh et al., 2016). The parasite’s life cycle mirrors that of *L. infantum*, with sand flies transmitting metacyclic promastigotes to humans (Fig. 1(b)). However, in AVL, transmission occurs exclusively through human-to-sand fly-to-human cycles, without an animal reservoir. This anthroponotic pattern has profound implications for control strategies, underscoring the need for early diagnosis and treatment to reduce the human infectious reservoir (Maia et al., 2018). Both species exhibit intracellular parasitism within macrophages, but their differing host reservoirs, canine for *L. infantum* and human for *L. donovani*, underscore the importance of tailored public health interventions in endemic regions through the reticuloendothelial system.

### 3.1 Pathogenesis

A variety of clinical and histological manifestations can be observed during leishmaniasis, directly reflecting the dynamic interaction between the parasite and the host immune response. These manifestations depend on the host’s immune response, and the state of the immune system strongly influences their intensity and outcome. Among immunocompromised patients, leishmaniasis can present with several serious and life-threatening manifestations, including atypical forms, disseminated infections, and inadequate healing in response to therapy, reflecting the immune system’s inability to inhibit intracellular parasite replication (Vechi et al., 2019; Yadav et al., 2023).

**Fig. 2:**
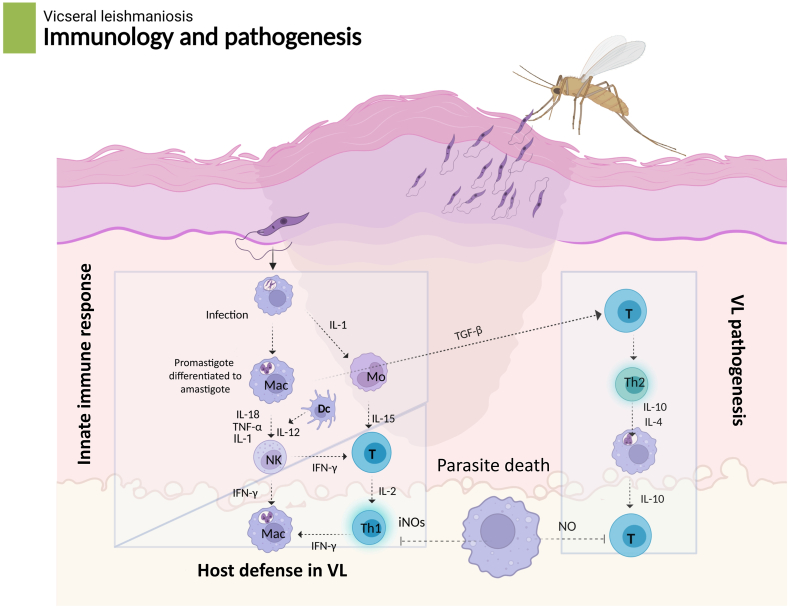
Immunology and pathogenesis of visceral leishmaniasis (VL). After being bitten by a sand fly, *Leishmania* promastigotes are injected into the skin, where they are phagocytosed by macrophages and undergo amastigote differentiation. The innate immune response is triggered by monocytes (Mo), dendritic cells (Dc), and macrophages (Mac). These cells generate cytokines, including IL-1, TNF-α, IL-18, and IL-12, which activate T cells and natural killer (NK) cells. The foundation of protective immunity in VL is formed by activated NK and Th-1 cells secreting IFN-γ, which improves macrophage effector capabilities and triggers the synthesis of inducible nitric oxide synthase (iNOs), which results in nitric oxide (NO)-mediated parasite death. In contrast, the development of Th2 responses and the generation of IL-4 and IL-10, which inhibit macrophage activation and enhance parasite survival, are linked to the progression of conditions. Additionally, TGF-β contributes to immunosuppression, tipping the balance toward VL pathogenesis. The outcome of infection is therefore determined by the balance between protective Th1-mediated host defense and detrimental Th2-mediated immune suppression. Created in https://BioRender.com.

The parasite can manipulate the host’s cell death mechanisms and immune response to ensure its survival during an infection (Bosurgi and Rothlin, 2021). In turn, the parasite alters phagolysosome maturation, modulates chemokine and cytokine production in host cells, and impairs their function, thereby directly attenuating macrophage activation and reducing parasite clearance. Through all these mechanisms, *Leishmania* enters the host cells and maintains successful differentiation within them (Carneiro and Peters, 2021). Disruption of cytokine balance, particularly in TNF-α and IFN-γ-dependent pathways, plays a critical role in disease progression and severe clinical outcomes. The pathological effects of TNF-α in co-infected patients may contribute to decreased cluster of differentiation 4 (CD4+) T-cell numbers, increased viremia, and increased seroconversion (Maksoud and El Hokayem, 2023; Okwor and Uzonna, 2013) (Fig. 2).

Because the host’s defense against *Leishmania* is mediated by T cells and their derived products, such as IFN-γ, which target infected macrophages, disruption of T-cell-dependent pathways can lead to an inability to control the parasite burden and to the transformation of subclinical infection into severe clinical disease (Jafarzadeh et al., 2022; Lodi et al., 2024). Diseases or drugs that alter these mechanisms may lead to severe visceral leishmaniasis, chronicity, and poor responses to treatment. This disease has been detected in patients with severely immunosuppressed conditions with defects of the T cells, such as patients who undergo kidney transplantation, patients with lymphoma and leukemia, patients who have systemic lupus erythematosus (SLE) or AIDS, and patients taking corticosteroids (Ramos et al., 2017).

*Leishmania* parasites can survive in macrophages by actively subverting host cellular defenses, allowing them to proliferate and replicate extensively within these cells (Fernandes and Zamboni, 2024). This intracellular persistence underlies VL pathogenesis in immunosuppressed patients and is associated with systemic parasite spread and severe clinical manifestations. In the following steps, cell bursting releases amastigotes, which are then absorbed by uninfected macrophages (Severino et al., 2022).

### 3.2 Immunology

T-helper cells (Th), especially the Th1 lineage, are effective at killing *Leishmania* protozoans in healthy, immunocompetent hosts (Clemente et al., 2018a). In addition, Th-1 cytokines (TNF-α, IFNγ, and IL-2) drive macrophages to activate and recruit to cells containing *Leishmania* amastigotes to phagocytose them (de Franca et al., 2024). In contrast, suppression of these responses in immunosuppressed patients leads to cellular immune dysfunction and exacerbation of the clinical manifestations of VL. There is a very high chance of developing clinical disease in patients who have immunosuppression, a condition that is characterized by inadequate T-cell responses and a disease course that is more serious and has a higher chance of relapsing in these patients. There is no doubt that HIV infection is a significant risk factor for developing VL, which is well established (Fletcher et al., 2015; Mohebali and Yimam, 2020). This immunodeficiency, characterized by a persistent decrease in CD4+ T-cells, is directly associated with the chronicity of the disease and a poor response to treatment. It has been found that when immunocompromised patients relapse, approximately 30% of them are found to have a CD4 lymphopenia (Costa et al., 2023b; Takele et al., 2023). Even though one of these patients has undergone treatment and was cured, it appears that their lymphocyte counts continue to be low, which could indicate that the lymphopenia was present before the onset of the VL.

It has been established that the length of time it takes for an infection to progress to an overt disease is dependent on several factors, including specific factors (immune status, nutritional status, age, genetic predisposition, and parasitic virulence) (Alvar et al., 2020). For the immune response against *Leishmania* to be effective, activated macrophages and the activation of a specific type of T-helper cell, type 1, are essential. Interestingly, a strong anti-leishmanial response involves proinflammatory cytokines that enhance the Th1 response, as well as active macrophage responses induced by IFN-γ and TNF-α. A mixed Th1/Th2 response is associated with the development of overt disease, and regulatory cells are believed to play a critical role in the immunodepression caused by VL (Pagliano and Esposito, 2017; Dayakar et al., 2019; Majoor et al., 2025) (Fig. 2).

Further, studies have shown that individuals with immunosuppressive conditions may develop and disseminate the infection, and that amastigotes may even be found in atypical locations, such as ocular tissues and the gastrointestinal tract (WHO, 2022; Yadav et al., 2023).

## 4 Detection of VL in immunocompromised individuals

Despite recent advances in diagnostic methods, diagnosing leishmaniasis remains a major challenge in the more remote regions of endemic countries. Furthermore, due to the intricate nature of its transmission cycle, which involves a wide variety of biological organisms, determining the species of *Leishmania* responsible for the infection is essential for controlling the disease and for implementing interventions (Hong et al., 2020). Previous studies have indicated that VL is often difficult to clinically diagnose in immunocompromised patients due to unusual manifestations and atypical anatomical features (Ceccarelli et al., 2018; Tolaj et al., 2023). A differential diagnosis should include disseminated mycobacterial infections, disseminated histoplasmosis, and lymphoma as potential causes (Pagliano and Esposito, 2017; Clemente et al., 2018a). There have been several reports describing VL as similar to, or mistaken for, an autoimmune disorder such as rheumatoid arthritis or SLE, or as a flare-up of the underlying disease (Mansoursamaei et al., 2025).

**Fig. 3:**
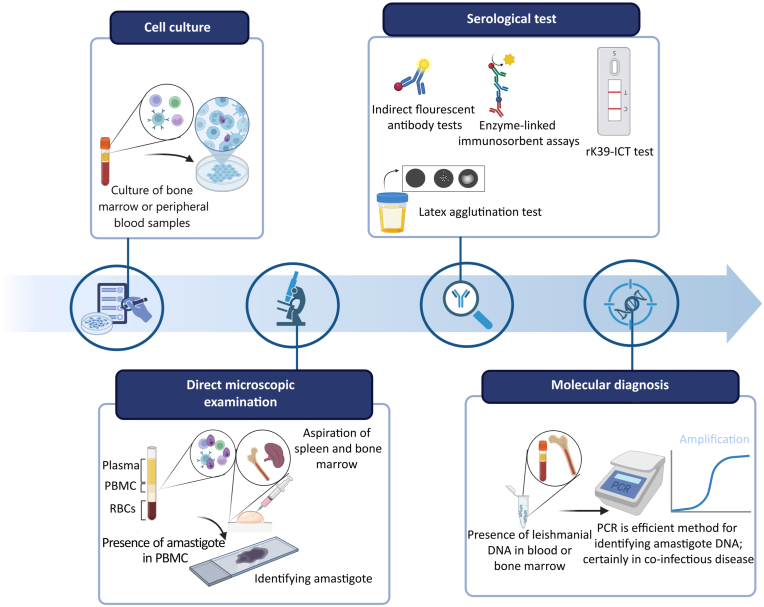
Diagnostic approaches for leishmaniasis. Several methods are employed to confirm *Leishmania* infection: Cell culture involves culturing bone marrow or peripheral blood mononuclear cells (PBMCs) to detect the presence of parasites. Direct microscopic examination remains the gold standard, requiring aspiration of spleen or bone marrow to visualize amastigotes within host cells. Serological tests detect anti-*Leishmania* antibodies in patient serum or urine, including indirect fluorescent antibody tests (IFATs), enzyme-linked immunosorbent assays (ELISA), rK39 immunochromatographic test (ICT), and latex agglutination test, which are widely used for field diagnosis. Molecular diagnosis, particularly polymerase chain reaction (PCR), identifies *Leishmania* DNA in clinical samples (blood or bone marrow) with high sensitivity and specificity, making it especially valuable in co-infections and cases with low parasite load. Each method varies in sensitivity, specificity, and feasibility, and a combination of approaches is often recommended for accurate diagnosis of leishmaniasis. Created in https://BioRender.com.

A stepwise diagnostic workflow for visceral leishmaniasis in immunocompromised patients is summarized below (Fig. 3). To ensure accuracy, diagnosis should be performed in a stepwise manner, accounting for the sensitivity and specificity of the methods across different patient groups. Methods include direct examination of spleen and bone marrow aspirates, serological tests, molecular diagnosis with PCR/qPCR, leishmanin skin test (LST), and parasitological culture. The sensitivity and specificity of each method vary depending on the patient’s immune status, type of immunodeficiency, and patient group. For example, PCR is considered one of the most sensitive and specific methods in HIV-infected patients, while serology is less reliable in these patients but has high sensitivity in solid organ transplant (SOT) recipients. This stepwise approach allows clinicians and researchers to increase the certainty of accurate diagnosis and reduce clinical errors by selecting the most appropriate diagnostic method for each patient, while providing information for designing a summary table or diagnostic workflow.

### 4.1 Direct examination

In immunocompetent individuals, the most commonly used diagnostic procedure for confirming the presence of VL is aspiration of the spleen and bone marrow, followed by direct microscopic examination (Gajurel et al., 2017; Lindoso et al., 2018) (Fig. 3). High parasitemia is uncommon in immunocompetent individuals and should raise suspicion of underlying immunosuppression.

**Fig. 4:**
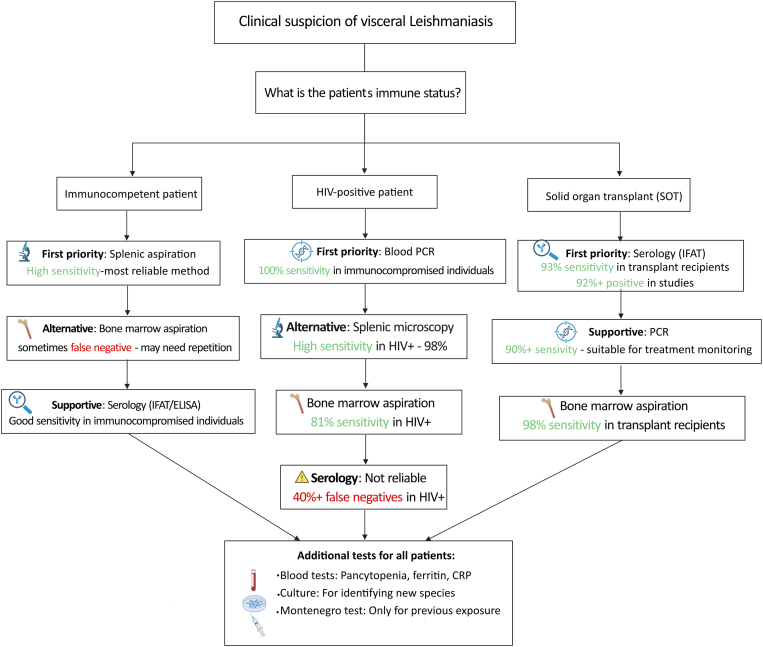
Diagnostic workflow for visceral leishmaniasis (VL) according to patient immune status. Splenic aspiration is most reliable in immunocompetent patients, blood PCR in HIV-positive patients, and serology in transplant recipients. Additional tests (blood counts, culture, Montenegro test) may support the diagnosis. Created in https://BioRender.com.

There is conclusive histopathological evidence for diagnosing VL when amastigotes are observed in a patient’s internal organs with clinical features relevant to the disease (Costa et al., 2023a; Lodi et al., 2024). Even though VL can affect multiple organs, as is evident at autopsy when the disease is disseminated, the diagnosis is usually made by direct microscopy of splenic aspirates or bone marrow to identify parasites (Heidari et al., 2019; Reimao et al., 2020a). Despite limited information on the sensitivity of direct microscopy of splenic aspirates in recipients of SOT, it has been shown to yield higher rates than bone marrow aspirates in a non-transplant setting and has been reported as positive even when the bone marrow examination was negative (Ramasubramanian et al., 2022; Muhsen et al., 2023). An aspiration of the spleen should not be performed as a first-line diagnosis because it is susceptible to intra-abdominal hemorrhage, particularly in thrombocytopenia (Abadías-Granado et al., 2021; Druzian et al., 2024). SOT recipients can also harbor amastigotes in liver tissue, but liver biopsy carries its own risks (Gajurel et al., 2017) (Fig. 4).

The presence of amastigotes in peripheral blood can also be detected (Diro et al., 2017). Based on a small study of immunocompetent and immunocompromised patients with VL, including transplant recipients, it has been shown that direct buffy coat examination had low sensitivity (66.7% and 58.8%, respectively) but 100% specificity (Gajurel et al., 2017).

Among those patients who are HIV-infected, the most sensitive means of diagnosis is through the use of microscopy on their spleen tissues (WHO, 2022). In HIV-positive patients, it was estimated that bone marrow microscopy had a sensitivity of 81% (Ivănescu et al., 2023). In contrast, the sensitivity was estimated at 98% for patients who have undergone a transplantation (Fig. 4). However, with a negative aspiration of bone marrow or a negative spleen finding, VL cannot be completely excluded, and several cases have been described in which the diagnosis of VL was confirmed only after an additional bone marrow examination (Gajurel et al., 2017).

### 4.2 Serology

Serology can also be positive even when the bone marrow aspiration does not show direct microscopy findings (Iatta et al., 2023; Baxarias et al., 2023). However, the specificity of serology as a means of diagnosing active infection can be challenging to determine, since some individuals may have been diagnosed with a pathogen, but their test may not have indicated ongoing pathogen replication due to their primary infection (Gajurel et al., 2017).

The diagnosis of VL in immunocompetent patients can be established using a variety of serological tests, including indirect fluorescent antibody tests and enzyme-linked immunosorbent assays (Mahshid et al., 2014; Ortalli et al., 2020; Akhtardanesh et al., 2021). Although many scientists believe that serological tests may complement other diagnostic tools, concerns remain about their cost-effectiveness, especially in financially impoverished countries (Cascio and Iaria, 2009). The serological tests performed on HIV-infected patients are clearly less reliable, as the levels of *Leishmania*-specific serum antibodies in those patients are much lower, and serological tests have shown that over 40% of those patients with VL and HIV co-infections are negative by serology as well (Botana et al., 2019; Voyiatzaki et al., 2024).

Although the diagnostic sensitivity is lower in HIV-infected patients, various studies have shown that serological tests have excellent diagnostic sensitivity when used in other groups of immunocompromised patients, such as those receiving SOTs or those with other immunocompromised conditions. The indirect fluorescent antibody test has been estimated to have a sensitivity of 48% when used in HIV-infected patients, while in transplant patients, it was estimated to have a sensitivity of 93% (Kumar et al., 2020; Ivănescu et al., 2023). Additionally, serology can be used to determine VL in patients currently receiving anti-TNF treatment (Botana et al., 2021).

Although many SOT recipients are often thought not to have the capacity to mount a robust immune response, in fact, *Leishmania*-specific serology can provide surprisingly accurate diagnostic results. In the study of 49 SOT recipients with VL, over 92% had a positive indirect fluorescent antibody test (IFAT), which was similar to historical cohorts that were HIV-negative, but was higher than historical cohorts that were HIV-infected (Gajurel et al., 2017) (Fig. 4)

rK39 immunochromatographic (rK39-ICT) is a rapid and commercial immunochromatographic test that can detect *Leishmania* antibodies at a quantitative level in serum (Leti Laboratories, Madrid, Spain) (Sarkari et al., 2018; Zhang et al., 2018; Mehrotra et al., 2025). Another option for diagnosing VL is to perform a latex agglutination test to detect leishmanial antigens in urine and confirm the infection. Although this test is non-invasive, rapid, and simple, the level of sensitivity in immunocompetent individuals is low to moderate (Fig. 3). In contrast, in HIV-infected patients, the sensitivity of this method can reach 85.7% over the first episode of the disease (Campos-Neto and Abeijon, 2020; Kumar et al., 2020).

### 4.3 Leishmanin skin test (or Montenegro test)

**Fig. 5:**
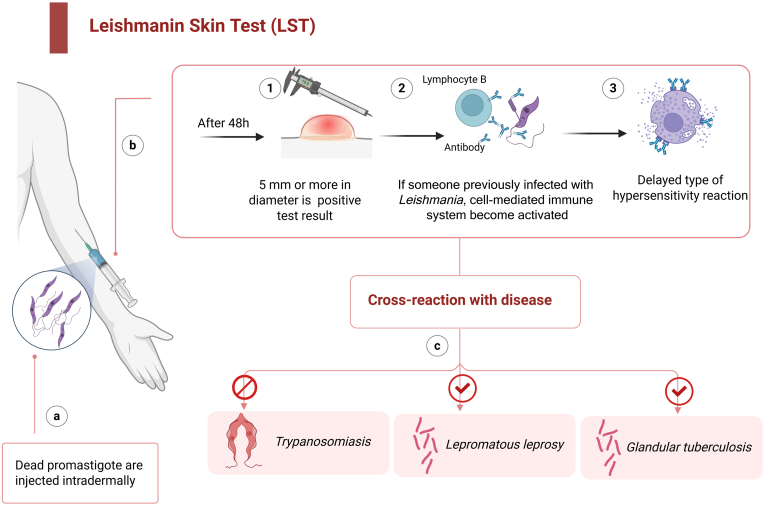
Leishmanin Skin Test (LST) and its immunological basis. The LST is an intradermal diagnostic test used to assess exposure or prior infection with *Leishmania.* (**a**) Dead promastigotes are injected intradermally into the forearm. (**b**) After 48 h, the site is examined: an induration of ≥ 5 mm is considered a positive reaction. A positive result reflects prior exposure to *Leishmania* and activation of cell-mediated immunity, leading to a delayed-type hypersensitivity response mediated by T lymphocytes and macrophages. (**c**) Although LST is useful in epidemiological studies, cross-reactions may occur with other granulomatous diseases such as lepromatous leprosy and glandular tuberculosis due to shared antigens. In contrast, *Trypanosoma* spp. infections do not produce cross-reactions because they lack antigenic similarity with *Leishmania*. Created in https://BioRender.com.

The leishmanin skin test (LST) (Montenegro test) measures delayed inflammatory reactions in the skin (Fig. 5 (b)) as a result of intradermal injection of a suspension of dead promastigotes and seems to be the only screening test that is capable of detecting asymptomatic *Leishmania* infections in the absence of symptoms (Fig. 5 (a)) (Guimarães et al., 2024). There is a belief that a positive LST result indicates that cell-mediated immunity has been established after asymptomatic infection or clinical remission of VL (Carstens-Kass et al., 2021). Positive LST results may appear months to years after infection. Nevertheless, it persists even after a positive result in an immunocompetent patient. In general, a positive LST result indicates that the patient has been exposed to *Leishmania* spp., and is thought to reflect a sustained cell-mediated immune response (Ivănescu et al., 2023). No cross-reactions were observed in patients with Chagas disease, but some were observed in patients with lepromatous leprosy or glandular tuberculosis (Skraba et al., 2015) (Fig. 5 (c)). In addition, prior treatment with immunosuppressive agents, which modulate the immune system *via* cell-mediated immunity, is likely to diminish the prognostic efficacy of LST (Alves et al., 2022). Since a skin test for leishmaniasis is a marker of prior exposure, it has little value for diagnosing active disease.

### 4.4 Molecular identification

It has been found that PCR tests can detect leishmanial nucleic acids in blood or bone marrow with high sensitivity (> 90%) in both immunocompetent and immunocompromised patients, compared with other methods such as culture, direct microscopic examination, and serology (Varani et al., 2017). A study reported that the PCR test for blood was 100% sensitive and specific when performed in patients with an immunocompromised state. However, a positive PCR result must still be evaluated within the appropriate clinical framework (Gajurel et al., 2017) (Fig. 4). To detect and quantify the DNA of *L. infantum*, real-time PCR (qPCR) was performed. Kinetoplast DNA was selected as the molecular target to achieve accurate sensitivity (Dantas-Torres et al., 2017; Mahajan et al., 2022). Specific pairs of primers (RV1 and RV2) were used as templates to generate the external primers and detect *Leishmania* parasites.

Molecular diagnosis by PCR is one of the most sensitive and specific methods for diagnosing VL in HIV-infected patients, and it is used in both peripheral blood and bone marrow samples as part of medical management. In addition, recent studies have indicated that serological, parasitological, and molecular tests for the detection of VL in HIV-positive individuals are just as practical as direct examinations of bone marrow aspirates in detecting the presence of this infection. PCR tests were also used in blood samples as a means to detect this infection in HIV-infected patients (Cota et al., 2012; Lindoso et al., 2018).

According to the most recent studies, PCR analysis of blood samples is the most efficient method for monitoring the effectiveness of long-term drug therapy and detecting relapses within a treated population, thereby reducing the need for repeated, more invasive procedures in the future (Hossain et al., 2017). The most common finding after a *Leishmania* infection is a negative PCR test obtained shortly after the start of treatment. In contrast, a positive PCR test result that recurs is a sign of relapse.

### 4.5 Culture media

In addition to microscopy, parasitological culture improves sensitivity; however, it is performed in only a limited number of laboratories and often delays diagnosis (Scarpini et al., 2022) (Fig. 3). Among the many culture media that may be employed to produce desired parasites, the Solid Novy-MacNeal-Nicolle (NNN) medium is the most frequently used for isolating and cultivating parasites. However, improvements were discovered when using a more nutritious medium (Hong et al., 2020). *In vitro* parasite development in culture media can take several days and may ultimately become contaminated with bacteria or fungi. Furthermore, depending on the parasite load in the biopsy sample, the experience of the laboratory operator, and the culture medium used, the findings may differ (de Oliveira Filho et al., 2024). As an alternative to cultivating parasites, a microculture approach has been developed over the past few years. The microculture of non-invasive samples, such as peripheral blood mononuclear cells or buffy coat, appears to have high sensitivity, and results may be obtained in as little as a few days or as long as two weeks (Scarpini et al., 2022). This approach is not only more sensitive and cost-effective than the conventional culture method, but it also does not require a needle and syringe. Additionally, it may be used to isolate parasites for drug-susceptibility evaluation and typing (Reimao et al., 2020a). Microscopy testing is estimated to be approximately 50% sensitive in detecting HIV infection based on blood smear staining results.

### 4.6 Laboratory diagnosis

The laboratory abnormalities are nonspecific and diverse. Besides pancytopenia, elevated acute-phase response proteins (ferritin, C-reactive protein, and sedimentation rate) are among the most common abnormalities (Fig. 4). The most common symptoms observed in patients were thrombocytopenia, splenomegaly, hepatomegaly, anemia, and leukopenia, which were observed in 85%, 75%, 42%, 86%, and 93% of patients, respectively (Gajurel et al., 2017).

It has also been observed that the coagulation cascade has been activated (an increase in D-dimer levels, a decrease in fibrinogen levels, a prolongation of the prothrombin time, and a prolongation of the activated partial thromboplastin time), circulating immune complexes, a decrease in complement levels, and cryoglobulinemia (Pasa et al., 2017).

The plasma protein profile in VL is characterized by polyclonal hypergammaglobulinemia, caused by the overactivation of B cells. The indirect Coombs test, as well as levels of anti-nuclear antibodies and anti-dsDNA antibodies, have been reported to be positive in patients who have VL, and these findings can lead to diagnostic errors (Sakkas et al., 2008). There is a wide variety of findings related to laboratory presentation found in the spectrum of HIV patients, patients with various rheumatologic conditions, and patients suffering from other immunosuppressive diseases without having signs of VL (Fontoura et al., 2018; Silva-Freitas et al., 2025).

## 5 VL and immunosuppressive viral disease

Human immunodeficiency virus (HIV) belongs to the family of retroviruses and is a double-stranded RNA virus. HIV is known to be the most common cause of immunosuppression historically (van Griensven et al., 2014). The human immune system is infected by members of this family, which, in turn, destroy or impair immune system cells. CD4+ T cells are primarily infected and replicated by HIV-1, causing depletion and a resultant immunodeficiency as a result (Becken et al., 2019; Masenga et al., 2023).

**Fig. 6:**
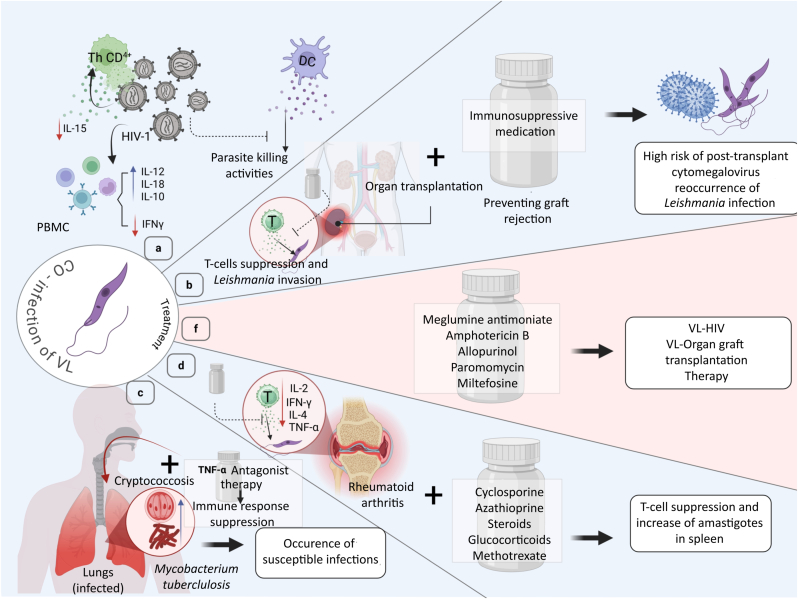
Visceral leishmaniasis (VL) co-infections with immunosuppressive conditions and their therapeutic challenges. (**a**) In HIV infection, CD4+ T cells are depleted, and dendritic cell (DC) function is impaired, leading to reduced IL-12 and IL-18 secretion. Peripheral blood mononuclear cells (PBMCs) show decreased IFN-γ and IL-15, while IL-10 is elevated, collectively suppressing Th1-mediated parasite killing and promoting *Leishmania* persistence. (**b**) In organ transplantation, long-term immunosuppressive therapy prevents graft rejection but predisposes to VL reactivation and post-transplant cytomegalovirus infection. (**c**) TNF-α antagonists impair macrophage activation, making people more vulnerable to opportunistic infections, including *Mycobacterium tuberculosis* and *Cryptococcus*. (**d**) In rheumatoid arthritis, immunosuppressants (cyclosporine, azathioprine, glucocorticoids, methotrexate) inhibit T-cell activity, promoting amastigote survival in the spleen. (**f**) The antileishmanial pharmaceuticals, which include miltefosine, meglumine antimoniate, amphotericin B, allopurinol, and paromomycin, continue to be the foundation of treatment for HIV and transplant patients. These treatments provide successful management of co-infections when accompanied by diligent monitoring and efforts to avoid recurrence. Created in https://BioRender.com.

Several studies have shown that the coexistence of *Leishmania* and HIV within a cell can affect the multiplication and expression of both species, as well as their interactions (WHO, 2022; Lodi et al., 2024). In Europe, where an overwhelming number of people with VL and HIV are intravenous drug users, compared to parasites found in the general population, the parasites responsible for the formation of VL in HIV-infected individuals show a greater level of polymorphisms in their enzymes. They are more diverse than those in the general population. Currently, there are some regions of East Africa where the level of co-infection between VL and HIV is highest, such as northwestern Ethiopia, where roughly 20–40% of those suffering from VL are also infected with HIV (Berhe et al., 2018; Grifferty et al., 2021). It is still unclear whether there will be any consequences from this interaction. Nevertheless, it has been suggested that *Leishmania* and HIV-1 can exploit dendritic cells (DCs) by modulating a range of cell-surface molecules, inhibiting DC function, producing soluble factors, delaying lysosomal fusion, and impairing intracellular killing (Getachew et al., 2020). The response of peripheral blood mononuclear cells (PBMCs) to *Leishmania* antigens has been demonstrated to be influenced by HIV infection. In HIV-infected patients, PBMCs stimulated with *Leishmania* antigen produce low levels of IFN-γ but high levels of IL-10. Additionally, the production of IL-12 or IL-18 (both of which are essential cytokines for the induction of robust Th1 responses) by PBMCs is lower in HIV-infected patients, VL patients, or VL/HIV co-infected patients compared with healthy donors (Okwor and Uzonna, 2013). Accordingly, these findings suggest that the inhibition of IFN-γ production by HIV and VL may not be solely due to the anti-inflammatory action of IL-10, but may also reflect the effects of HIV on the regulation of IFN-γ-inducing factors, such as IL-12 and IL-18. Accordingly, studies have shown that patients who are HIV-infected alone produce higher amounts of IL-10 and IL-4 than those who are co-infected with VL and HIV (Costa et al., 2025) (Fig. 6 (a)).

In addition, another immune cytokine, IL-15, appears to be modulated by *Leishmania*/HIV co-infection. IL-15 enhances CD8+ T-cell responses and the production of cytokines that enhance our protection against intracellular parasites. There has been a report that HIV-infected immunocompromised patients have a low level of IL-15 in their serum (Maksoud and El Hokayem, 2023). These cytokine abnormalities, including decreased IFN-γ, IL-12, and IL-18, and increased IL-10 and IL-4, directly inhibit the function of dendritic cells and macrophages in antigen presentation and in the production of proinflammatory cytokines. As a result, Th1 responses are reduced, and parasite and virus control is impaired, which together contribute to faster disease progression and reduced efficacy of antiviral and antiparasitic therapies. Infected macrophages become a breeding ground for parasite and virus proliferation by altering signaling pathways and reducing proinflammatory cytokine production (Fig. 2). This framework of cytokine abnormalities and cellular dysfunction suggests important clinical consequences, including treatment resistance, chronic infection, and the need for combination therapy.

Even though HIV and *Leishmania* have an additive pathogenic effect on dendritic cells and macrophages, the drugs that are widely used to treat HIV are unable to minimize the infection rate of *Leishmania*, regardless of their ability to increase immunity and reduce proliferation (WHO, 2022). Supplementary Table S1 summarizes the previous studies on the epidemiology of visceral leishmaniasis and HIV coinfection.

## 6 VL and immunosuppressive infectious disease

Immunosuppressive disorders that are not correlated with HIV are becoming more common in the modern era. This is mostly due to improved medical care for patients with chronic illnesses and the therapeutic use of immunosuppressive medicines (Colomba et al., 2020). This group comprises heterogeneous medical conditions that hinder the immune system’s ability to manage VL and either increase the reactivation of a dormant infection or impair the control of a recently acquired infection (Lodi et al., 2024). These disorders, which can affect multiple immune system layers, are commonly observed in transplant, oncologic, or rheumatologic patients because they can affect T-cell lymphocytes through mechanisms such as cell cycling, maturation interference, depletion, co-stimulation, and tolerance induction (Lodi et al., 2024). Mechanistically, many immunomodulatory therapies directly disrupt key intracellular control pathways of *Leishmania*. TNF-α inhibitors reduce parasite-killing capacity by suppressing macrophage activation and inhibiting nitric oxide and reactive oxygen species production. Targeted therapies against IL-6, janus kinase/signal transducer and activator of transcription (JAK/STAT), or CD20 can also disrupt the balance of Th1/Th2 responses, providing a platform for parasite proliferation by attenuating IFN-γ production and disrupting T-cell-macrophage interaction (Igbokwe, 2018; Lafleur et al., 2024). In hematological malignancies, in addition to neutropenia and lymphopenia, qualitative impairment of T- and B-cell function and reduced antigen presentation by dendritic cells are observed, leading to an inability to suppress primary infection or prevent reactivation of latent infection (Bogdan, 2012; Al-Kamel, 2017). This convergence of cellular and cytokine defects explains the higher susceptibility of these patients to invasive and recurrent VL. There has been an increase in the prevalence of non-HIV-related VL in countries that are not tropical (Lodi et al., 2024). This may be ascribed not only to the growing number of patients who are suffering from chronic illnesses but also to the rapid development of immune-modulating treatments for the treatment of neoplastic disorders. Since 1988, research has recorded many instances of VL in individuals who were diagnosed with lymphoproliferative diseases (Banegas et al., 2024). These findings indicate the presence of VL in a number of lymphoproliferative disorders, including splenic marginal zone lymphoma (SMZL), classic Hodgkin lymphoma, follicular cell lymphoma, chronic lymphocytic leukemia, lymphoplasmacytic lymphoma, T-cell prolymphocytic leukemia, and angioimmunoblastic T-cell lymphoma (Kalmi et al., 2020; Banegas et al., 2024; de Caceres et al., 2026). VL was misclassified as a progression of the underlying hematological malignancy in the majority of cases (at least 8 of the 12) (Agura, 2024). Available epidemiological data indicate that the incidence of VL in patients receiving biological therapies or with hematological malignancies is significantly higher than in the general population in the same regions, especially in Mediterranean countries. Reports suggest a several-fold increased risk of VL development or reactivation in patients receiving TNF-α inhibition and in hematopoietic cell transplant recipients (Lodi et al., 2024; Moura de Souza et al., 2025). However, the lack of large cohort studies and standardized comparative data limits accurate estimates of absolute and relative risks and makes it difficult to interpret the true burden of disease. Although this cannot be proven with certainty, VL occurring in non-endemic areas may result from either a recent acute infection after traveling to endemic countries or from parasite reactivation during episodes of immune system suppression, such as following hematopoietic cell transplantation; this is illustrated by a case involving concomitant VL, graft failure, and lymphoma relapse (Banegas et al., 2024). Despite studies showing a reduction in relapses, there is still insufficient evidence on screening for VL in candidates for immunosuppressive treatment or organ transplantation in endemic regions. Furthermore, guidelines for secondary prevention in non-HIV-immunosuppressed VL individuals lack clarity (Lodi et al., 2024). In a case series study, it has been highlighted that discontinuing TNF-α antagonist therapy for patients with tuberculosis (TB) and pulmonary cryptococcosis resulted in paradoxical clinical reactions that were attributed to the reconstitution of the immune system (Fong, 2020; Armange et al., 2023) (Fig. 6 (c)).

Despite the increase in reported cases, screening strategies before starting immunosuppressive therapies in endemic areas lack international coherence and consensus. Currently, there are no specific and standardized guidelines for routine VL screening in patients who are candidates for biological therapies or organ transplantation, and recommendations are largely based on case reports and expert opinion. Furthermore, in the area of secondary prevention, clear criteria for the duration and type of monitoring or maintenance therapy in non-HIV-infected immunosuppressed patients have not been defined, which increases the risk of relapse, late diagnosis, and mortality. These policy gaps indicate the need for prospective studies and the development of evidence-based guidelines for screening and secondary prevention in these high-risk populations.

## 7 VL and organ transplant recipients

Solid organ recipients have generally developed latent infections during the course of immunosuppressive therapy, although it is not yet clear how an individual medication can reactivate latent infections or contribute to the progression of asymptomatic infections.

Among patients receiving immunosuppressive therapy to prevent graft rejection, this affects T cells, altering their ability to defend against intracellular microorganisms, such as *Leishmania*, and thus increasing their risk of developing manifestations of the disease (Akuffo et al., 2018; Favi et al., 2022). Despite this, the exact mechanism underlying VL risk remains unclear. There is no correlation between the development of VL in recipients of SOT and the patient’s socio-economic status, the origin of the organ, or the type of transplant, although VL is more common in patients who have received kidney transplants, as these are the most commonly performed transplants (Busutti et al., 2023; Viana et al., 2025). Rather, it depends much more on the degree of exposure to infection. SOT patients have a 135-fold greater risk of developing VL than immunocompetent individuals living in the same area (Akuffo et al., 2018; Clemente and Mourão, 2020). Several cases have been reported in which patients with CL or VL before the transplant developed recurrent disease after post-SOT. The incidence of relapsed VL was detected in 24–35% of SOT patients as early as one month after transplantation, and as late as five years after transplantation (Clemente et al., 2018a).

There is evidence that post-transplant cytomegalovirus infection can be associated with the development of VL in kidney transplant recipients (Imen et al., 2021; Busutti et al., 2023). However, other studies have not found any association between the infections and VL in patients receiving SOT (Clemente et al., 2015). In a study of patients who have undergone kidney transplants, it was found that living with certain animals, especially cats, is a significant risk factor in countries with an endemic disease (Alves da Silva et al., 2013; Gajurel et al., 2017) (Fig. 6 (b)). Overall, clinical findings appear similar to those of HIV-infected and immunocompetent patients with VL; however, hepatosplenomegaly occurs less frequently in SOT recipients, and leukopenia is more prevalent than in HIV-negative patients with historical VL. In a study of 30 kidney transplant recipients with VL, it was found that fever was present in only 70% of the patients, whereas all of the patients suffered from weight loss, as well as splenomegaly and hepatomegaly, which were observed in 93% and 70% of those patients, respectively (Gajurel et al., 2017).

There has been evidence that VL can also affect patients who have had transplanted kidneys, causing the kidney graft to fail, which requires some patients to be on dialysis as a result of their condition. This can occur in approximately 75% of patients, even when the infection has been cured (Busutti et al., 2023). Often, patients with VL, particularly those who have undergone transplants, have other pathogens in their bodies, which can lead to confusion in diagnosis and delays in the appropriate management of patients (Gajurel et al., 2017).

In most cases, VL occurs following a transplant as a late complication, with a median delay ranging between 6 and 19 months, depending on the organ transplanted (6 months for a liver transplant compared to 19 months for a kidney transplant) (Pagliano and Esposito, 2017). As a result of a low index of suspicion or misdiagnoses, a diagnosis of this disease can be delayed for several months in transplant recipients (e.g. drug toxicity, which in some cases led to the discontinuation of azathioprine)

## 8 VL and cancer

Cancer is known as a variety of disorders characterized by the abnormal proliferation of cells that tend to multiply in an unregulated process and, in some situations, metastasize. Cancer can affect almost all tissues in the human body. It is the second most common cause of mortality in humans, behind coronary artery disorders. Every year, the number of new instances of cancer that are recorded exceeds around 19.3 million, while the number of deaths caused by cancer that occur throughout the world is estimated to be 10 million (Zuchowska and Skorupska, 2022). The clinical symptoms of cancer may vary extensively, and immunosuppression is a significant side effect that must be taken into consideration while managing cancer patients (Rashidi et al., 2021a, Rashidi et al., 2021b). However, viral, bacterial, and parasitic infections have been linked to human malignancy for many years. The following are examples of possible connections between malignancy and leishmaniasis that have been identified: (i) leishmaniasis imitating cancer; (ii) co-occurring leishmaniasis and cancer; (iii) cancer growing in leishmaniasis patients; and (iv) leishmaniasis developing in malignant patients (Al-Kamel, 2017; Mohaghegh et al., 2024).

In addition, leishmaniasis causes chronic inflammation and affects the activation and function of dendritic cells and macrophages. It is interesting to note that persistent inflammation causes cancer *via* a variety of processes, including DNA damage and genomic instability, which may result in genetic and epigenetic changes that initiate cancer (Ibrahim et al., 2023). Furthermore, a variety of malignancies exploit the immunomodulatory properties of cytokines, which can influence gene expression and suppress immune responses designed to eradicate cancerous cells. Therefore, many tumor cells release the immunosuppressive cytokine IL-10, which is accompanied by a simultaneous reduction in the ability to stimulate protective Th1 responses. As a result, the Th2 microenvironment and the persistent inflammation seen in apparent leishmaniasis may potentially contribute to the development and advancement of cancer, and *vice versa* (Schwing et al., 2019; Rahim et al., 2025).

A considerable body of research has shown a well-established association between *Leishmania* spp. and the pathogenic process underlying malignant lesions, including leukemia, lymphoma, hemangiosarcoma, basal cell carcinoma, and squamous cell carcinoma (Rashidi et al., 2021a, Rashidi et al., 2021b). There are occasions when the clinical parameters and physical characteristics of CL and VL closely resemble those seen in a variety of neoplasms. For instance, the signs and symptoms of lymphoma, which include weight loss, fever, chills and sweating, acute exhaustion, swollen belly, and enlarged lymph nodes, bear a striking resemblance to those that are noticed in VL. Subclinical forms of VL were misidentified as adrenal cystic masses, cutaneous spindle-cell pseudotumors, nasopharyngeal tumors, lymphomas, and histiocytoses (Schwing et al., 2019).

*Leishmania* parasites have been the subject of numerous studies investigating their potential effects on anticancer immunity and their role in regulating the host’s immune system. A number of studies have shown that *Leishmania* parasites can disrupt the host’s chromatin structure, potentially altering immune-related genes and responses (Lecoeur et al., 2020; Brar et al., 2022; Machado and Moretti, 2026). The role of immune checkpoint molecules in modulating immune responses is a significant similarity between leishmaniasis and cancer. Certain species of *Leishmania* induce immune checkpoint molecules, such as Cytotoxic T-lymphocyte-associated protein 4 (CTLA-4), which can impair anti-parasitic immunity and contribute to the course of the disease (Rashidi et al., 2021a, Rashidi et al., 2021b; Costa-Madeira et al., 2022; Tamir et al., 2025). There has been a strong association between VL and the administration of various chemotherapeutic agents and monoclonal antibodies in patients with malignancies; most cases have been reported in patients with hematologic malignancies (Al-Kamel, 2017; Neto et al., 2024). Additionally, chemotherapies that are delivered against some types of cancer are associated with immunosuppression and/or immune dysfunctions. An example of this is the proteasome inhibitor bortezomib, which reduces DC activity, the number of T lymphocytes, and IFN-γ production. On top of that, molecules produced by parasites, such as heat shock protein 100 (HSP100), play significant roles in parasite survival and in interfering with immune system responses. These functions are comparable to those that similar molecules play in the survival of cancer cells (Rashidi et al., 2021a, Rashidi et al., 2021b; Prasanna and Upadhyay, 2021). Because of these similarities, researchers can develop common treatment strategies for both diseases. Additionally, proteins such as HSP90 and HSP60, which are essential for cell integrity and survival, play a key role in both leishmaniasis and cancer. Inhibitors of these proteins have shown promise not just in the treatment of cancer but also in the management of leishmaniasis (Rahim et al., 2025).

The effectiveness of compounds originally developed for cancer treatment has been demonstrated against *Leishmania*. Miltefosine, for instance, was first developed as an anticancer agent; yet it has only recently become the first oral medicine licensed for the treatment of leishmaniasis (Reimao et al., 2020b). This highlights that both diseases share similar mechanisms. Furthermore, topoisomerase I inhibitors, such as camptothecin, which are often used in cancer treatment, are effective against *Leishmania donovani* topoisomerase. This underscores the therapeutic overlap and the possibility of medication repurposing (Rahim et al., 2025).

Th1-type immune responses, IFN-γ production, and macrophage activation are vital components of effective control of *Leishmania*; however, these pathways are often disrupted by cancer itself and by anticancer treatments (Yadagiri et al., 2023; Paton et al., 2025). Cancer-associated immunosuppression has been documented to expose patients to intracellular organisms, suggesting that VL emerges or reactivates in these situations, albeit often criminally undershot compared to other opportunistic infections, a phenomenon also observed with anti-cancer treatments (Bogdan, 2012).

VL has been documented to show clinical and other susceptibility measures that align with a disease state of cancer, and also garner, in some instances, VL and cancer, a prevailing and shared bottom-up process of documented immunological and possible molecular pathways that interface with both disease states, and also suggest a possibility of translational value (Shafiei et al., 2014). For example, macrophage activation, along with JAK/STAT signaling and some immune checkpoints, particularly programmed cell death protein 1 (PD-1) and programmed death-ligand 1 (PD-1/L1), are mechanisms that prospectively interface with antitumor immunity and host defense mechanisms against *Leishmania* (Jafarzadeh et al., 2022; Hadifar et al., 2024). These interactions show the potential for VL and cancer to recycle advances made available for immunocancer treatment, and for immunocancer treatment to recycle advances made available in the treatment of VL (Rashidi et al., 2021a, Rashidi et al., 2021b). Identifying similar therapeutic targets can enhance collaboration between Parasitology and Oncology disciplines, moving from description to translational research by providing clinically relevant evidence on the effects of immunosuppression on VL susceptibility and severity (Roatt et al., 2014).

## 9 VL and autoimmune diseases

The development of VL has been linked to the use of multiple immunosuppressants prescribed for rheumatism, including cyclosporine, azathioprine, steroids, glucocorticoids, and methotrexate (van Griensven et al., 2014; Ramos et al., 2017; Nath et al., 2021). Furthermore, a higher incidence has also been reported among people on modern immunosuppressant drugs, such as TNF-α antagonist (Bosch-Nicolau et al., 2019; Kurizky et al., 2020). By inhibiting cytokine expression, glucocorticoids alter the functions of T cells as suppressors, effectors, and cytotoxic agents, thereby increasing susceptibility to infection. In a murine model, prolonged steroid use can reduce the production of IL-2, IFN-γ, IL-4, and TNF-α, and can increase the amastigote burden in the spleen by 3-fold (Pagliano et al., 2016).

In the past few years, there have been several reports indicating that people taking anti-TNF therapy have been diagnosed with VL (De Leonardis et al., 2009; Guarneri et al., 2017; Catala et al., 2015; Horrillo et al., 2019). However, the increased susceptibility for the development of VL in such cases may be consistent with a theory regarding immunity against *Leishmania* based on TNF activity, but we cannot rule out the possibility that the higher risk of developing VL can be attributed to other factors associated with the rheumatological disease itself (Pagliano et al., 2016).

According to experimental studies, using drugs that suppress the immune system, such as methotrexate and anti-TNF agents, decreases T cell responses that drive inflammation. The use of those immunosuppressive drugs also increases the number of inhibitory signals in the immune response, such as PD-1 and IL-10, leading the immune system not to clear parasites as it would in a healthy immune system (Bernardo et al., 2023). Immunosuppressive drugs make patients more likely to get VL and/or suffer from more severe VL-related diseases (Bernardo et al., 2021).

Such dysfunctional cytokine profiles mirror the clinical observation that patients receiving long-term immunomodulatory drugs for autoimmune conditions have a higher risk of developing VL or experiencing more severe disease, likely due to compromised Th1 responses, which are critical for controlling *Leishmania* proliferation (Schwartz et al., 2019; Menon and Elias, 2020). Moreover, impaired immune effector function in these contexts has important clinical implications, underscoring the need for early screening, vigilant monitoring of latent or asymptomatic infection before initiating immunosuppression, and carefully tailored treatment protocols (e.g. prolonged antiparasitic therapy, secondary prophylaxis) to mitigate the increased risk and improve outcomes in immunosuppressed individuals (Bernardo et al., 2023; Gomez and Weese, 2024) (Fig. 6 (d)).

Cytokine assays have been successfully used to detect asymptomatic *Leishmania* infection in patients receiving immunosuppressive treatment for autoimmune diseases, as well as to assess cure in patients requiring treatment for VL (Botana et al., 2021)

## 10 Treatment of VL in immunosuppressed patients

Immunosuppression is one of the greatest risk factors for the development of overt disease, as it can adversely affect the appearance and response to treatment (van Griensven and Diro, 2019). The most difficult obstacle in improving the outcomes for co-infected patients is their late presentation (Akuffo et al., 2018). There are several treatments available for VL, including pentavalent antimonials (meglumine antimoniate or sodium stibogluconate) and formulations of amphotericin B (de Souza et al., 2020; Zhang et al., 2025) (Fig. 6 (f)).

Regarding the treatment of VL among HIV-positive patients, the majority of drugs have not proven particularly effective. There is a significant association between HIV co-infection and higher relapse and failure rates in treatment (Akuffo et al., 2018; Mohammed et al., 2020). Furthermore, there is an increased likelihood of drug toxicity and the number of deaths associated with treatment. With antimonials in particular, there are a number of studies that have shown increased toxicity to patients with HIV co-infection (Cota et al., 2014; Lindoso et al., 2018). This threat can be life-threatening in some cases. Repeat episodes of VL tend to present difficulties for HIV-infected patients and often exert a negative impact on the recovery of their CD4 levels. Therefore, it is of utmost importance to prevent VL relapse from occurring (van Griensven et al., 2014).

Non-HIV immunosuppressive conditions have a higher initial treatment response rate and a lower rate of recurrence than HIV-infected individuals (Lindoso et al., 2018; Silva-Freitas et al., 2025). However, the results are not as good as those seen among immunocompetent individuals. Among transplant recipients, there was an initial cure rate of 84%, which is significantly higher than the cure rate reported among HIV-positive patients (55–66%), although it was not as high as the cure rate that was reported among immunocompetent patients (93–97%) (Antinori et al., 2008; Gajurel et al., 2017).

In areas where VL is prevalent, it should be considered along with other opportunistic infections, and up to 20% of VL-HIV co-infected patients are detected as being infected with TB. The highest mortality risk is associated with these patients, as well as therapeutic challenges regarding initiation of (or continuation of) TB, VL, and HIV treatment simultaneously (Akuffo et al., 2018). WHO states that 40 mg/kg of liposomal amphotericin B is considered the best treatment for patients with VL-HIV. Despite modest efficacy and acceptable toxicity profiles, 30 mg/kg miltefosine dosed for 28 days seems to have a promising efficacy profile, with an initial cure rate of 81% in HIV co-infected patients (Adriaensen et al., 2018). The oral drug miltefosine, which has recently been introduced to the market as an option for treating VL, should be investigated in patients with immunocompromised systems (van Griensven et al., 2024). The results of several studies have demonstrated that this drug, which has successfully been prescribed to Indian patients suffering from Kala-Azar, has shown the ability to be used in immunocompromised individuals as a relapse prevention or treatment drug (Datta et al., 2021; Sundar et al., 2024).

A total dose of 21 mg/kg of amphotericin B is recommended for the treatment of VL in transplanted patients, as it is for immunocompetent individuals (Clemente et al., 2018b); however, other treatments, such as antimonial and allopurinol, have also been tried with varying degrees of success (Ejaz et al., 2014). The use of lipid formulations of amphotericin B (L-AmB) for the treatment of VL is becoming increasingly common worldwide due to their better tolerability compared with amphotericin B deoxycholate (AMB-D), which is thus becoming the treatment of choice for this disease. Exact dose and duration of treatment with L-AmB have not been established; however, based on case reports and HIV guidelines, the recommended dose of L-AmB is 4 mg/kg IV daily for 5 days, followed by weekly for 5 weeks, depending on the patient (Sundar and Chakravarty, 2015; WHO, 2022). Sodium stibogluconate should be administered intravenously or intramuscularly for 28 days at a dose of 20 mg/kg each time (Sundar and Agarwal, 2018). Other agents have also been reported to be successful, including allopurinol, either alone or in combination with other medications (fluconazole or ketoconazole), or paromomycin (Kotb Elmahallawy and Agil, 2015; Ham and Temesvari, 2020; Kasabalis et al., 2020).

Most endemic countries recommend nutritional supplementation before initiating VL therapy, and this is also recommended by the WHO and national VL guidelines. The use of ready-to-use therapeutic foods for the control of TB and HIV can be found in some hospitals around the world. However, because VL is still perceived as a neglected disease, the majority of these patients do not receive nutritional therapy as part of their treatment for VL (Akuffo et al., 2018).

Infections sustained by intracellular agents (e.g. *Listeria monocytogenes*) can be difficult to treat in immunocompromised HIV-negative individuals. Compared to HIV positives, relapse and response rates are higher, but not as high as in immunocompetent individuals. Several authors have recommended using L-AmB in these cases due to its high safety profile (Paila et al., 2010; Pagliano and Esposito, 2017; Wasan et al., 2022). When considering the mechanisms by which Th1 response impairment and macrophage activation defects affect intracellular parasite clearance, it is clear that they limit the efficacy of antileishmanial medications (Lodi et al., 2024). There is evidence for the efficacy of combination or sequential therapy among at-risk immunosuppressed patients; for example, L-AmB followed by miltefosine or periodic low-dose L-AmB prophylaxis will decrease relapse rates without significantly increasing toxicity (Pérez-Jacoiste Asín et al., 2017; Burza et al., 2022). Upon finding this information, the need for more aggressive subgroup-specific treatment approaches has been demonstrated and is warranted, rather than the uniform approach of using only unilateral therapy in patients with immunodeficient disease (Pagliano and Esposito, 2017; Lindoso et al., 2018).

Solid organ transplant patients who are not infected with HIV receive an initial cure rate of 70–88% for VL when treated using L-AmB; however, they also experience a relapse rate of 15–25% following their initial cure. The relapse rate in HIV-positive patients (> 50%) is higher than that of SOT patients (Lindoso et al., 2018). Patients with hematological malignancies have been reported to have an elevated relapse (approximately 30–40%) and treatment-related mortality rate, as a result of bone marrow impairment and the effects of cytotoxic therapies (Sellitto et al., 2022). Thus, patients with hematological malignancies may require extended or repeated therapies to eliminate residual intracellular parasites, as their immune systems remain impaired. These discrepancies further highlight that the degree of immune impairment and its reversibility determine the efficacy of VL treatment (van Griensven and Boelaert, 2011; Pagliano and Esposito, 2017). The first-line, alternative, and adjunct therapies stratified by immunosuppressive condition are summarized in Table 1.

**Table 1:** First-line, alternative, and adjunct therapies stratified by immunosuppressive condition.

Immunosuppressive condition	First-line therapy (recommended)	Alternative options (when first-line therapy not available/tolerated)	Adjunct therapy & maintenance (prevent relapse/support)
HIV/AIDS (CD4+ < 200 cells/μl)	Liposomal amphotericin B (L-AmB): total dose 40 mg/kg; Days 1–5, 10, 17, 24, 31, 38; OR 3–4 mg/kg/day × 10 days	Miltefosine 2.5 mg/kg/day × 28 days (max 150 mg/day); initial cure: ∼81%; better in combination	Secondary prophylaxis MANDATORY; monthly L-AmB: 3–4 mg/kg; continue until CD4+ > 350 × 6 months
	Initiate ART within 2 weeks if ART-naïve	L-AmB + Miltefosine (short-course combination)	Alternative: Pentamidine: 4 mg/kg monthly (if L-AmB unavailable)
	Expected cure: 55–66%	Paromomycin 15 mg/kg/day × 21 days (limited evidence)	Monitor: PCR, clinical assessment
			Reduces relapse from 60% to 20–30%
Solid organ transplant recipients	L-AmB: total dose 21–24 mg/kg (same as immunocompetent); 3 mg/kg/day × 7 days; OR Days 1–5, 10, 17, 24, 31, 38	Amphotericin B deoxycholate (AmB-D): 0.75–1.0 mg/kg/day; total 15–20 mg/kg; higher nephrotoxicity risk	Maintenance if relapse occurs (not routine for first episode)
	Monitor drug-drug interactions with immunosuppressants	Sodium stibogluconate: 20 mg/kg/day × 28 days IV/IM (use with caution - cardiotoxic)	Monthly L-AmB: 3 mg/kg × 6–12 months if relapse history
	Expected cure: ∼84%	Allopurinol: 20 mg/kg/day + Azole (experimental)	PCR monitoring recommended
			Adjust immunosuppression (reduce if possible)
			Relapse rate: 24–35%
Autoimmune & rheumatologic diseases	L-AmB (standard dosing): 21–24 mg/kg total dose	Sodium stibogluconate: 20 mg/kg/day × 28 days (if NOT on TNF-α inhibitors)	Resume immunosuppression AFTER documented cure
	Pause/reduce TNF-α inhibitors and other biologics during Rx	Miltefosine: 2.5 mg/kg/day × 28 days	Monitor for relapse: Clinical assessment every 3 months × 1 year. Consider PCR follow-up
	Continue corticosteroids (reduce if possible)	Meglumine antimoniate: 20 mg/kg/day × 28 days	Restart biologics cautiously
	Expected cure: 70–85%	Pentamidine: 4 mg/kg every other day × 15 doses	Relapse rate: 20–40%
Hematologic malignancies (leukemia, lymphoma)	L-AmB (dose-adjusted): 3–4 mg/kg/day if ANC > 500; 1–2 mg/kg/day if neutropenic; extended duration often needed	AmB-D (if L-AmB unavailable): 0.5–0.7 mg/kg/day in neutropenia	Prophylaxis during chemotherapy: L-AmB 3–4 mg/kg monthly OR pentamidine 4 mg/kg monthly
	Coordinate with oncology for chemotherapy timing	Combination therapy: L-AmB + Miltefosine (better outcomes reported)	Extended treatment duration (> 4 weeks often required)
	Expected cure: 70–80%	Pentamidine (salvage only)	G-CSF support as needed
		Avoid antimonials during chemotherapy (additive toxicity)	Monitor: CBC, PCR, clinical status
			High relapse risk with ongoing chemotherapy
Other immunosuppressors (corticosteroids, etc.)	L-AmB (standard dosing): 21–24 mg/kg total dose	Antimonials (if steroid dose can be reduced to < 20 mg/day; prednisone equivalent)	Monitor for relapse during immunosuppression escalation
	Reduce corticosteroid dose if medically feasible	Miltefosine	Consider maintenance if: prolonged high-dose steroids; multiple relapses
	Expected cure: 75–90%	AmB-D	Resume monitoring if immunosuppression intensifies

*Abbreviations*: ANC, absolute neutrophil count; AmB-D, amphotericin B deoxycholate; ART, antiretroviral therapy; CBC, complete blood count; CD4+, CD4+ T lymphocytes; G-CSF, granulocyte colony-stimulating factor; IM, intramuscular; IV, intravenous; L-AmB, liposomal amphotericin B; PCR, polymerase chain reaction; TNF-α, tumor necrosis factor-alpha.

## 11 Prevention of VL in immunosuppressed patients

There is no doubt that living in an endemic area is the greatest risk factor for VL infection (Hassan et al., 2022). Males are more likely to be affected by this illness than females, possibly because they are exposed to more insect vectors (Motuma et al., 2016; Duarte et al., 2025). As of now, no vaccine is available for general use (Elikaee et al., 2019; Selvapandiyan et al., 2024). Among the best ways to prevent the spread of this disease is to control vectors and avoid exposure to sand flies (for instance, by wearing long-sleeved clothing in areas where the disease is prevalent). Nevertheless, it is worth noting that the known geographical niches of sand fly vectors may expand with a changing climate, leading to the development of new areas of endemicity (Curtin and Aronson, 2021).

Similarly, international travel and migration can also affect leishmaniasis transmission, as introducing the disease into new geographical areas can provide local vectors with a vehicle for maintaining autochthonous transmission. It has been reported that recipients of SOT have acquired leishmaniasis during their travels to areas where the disease is endemic (Gajurel et al., 2017; Clemente et al., 2018a). Unfortunately, over the past few years, there has been an alarming increase in the number of leishmaniasis cases among displaced refugees from endemic areas that have been affected by conflict (Du et al., 2016; Tarnas et al., 2024). Over the last several years, an increase in leishmaniasis cases among organ transplant recipients has been attributed to a combination of increased migration and foreign travel (Gajurel et al., 2017).

**Fig. 7:**
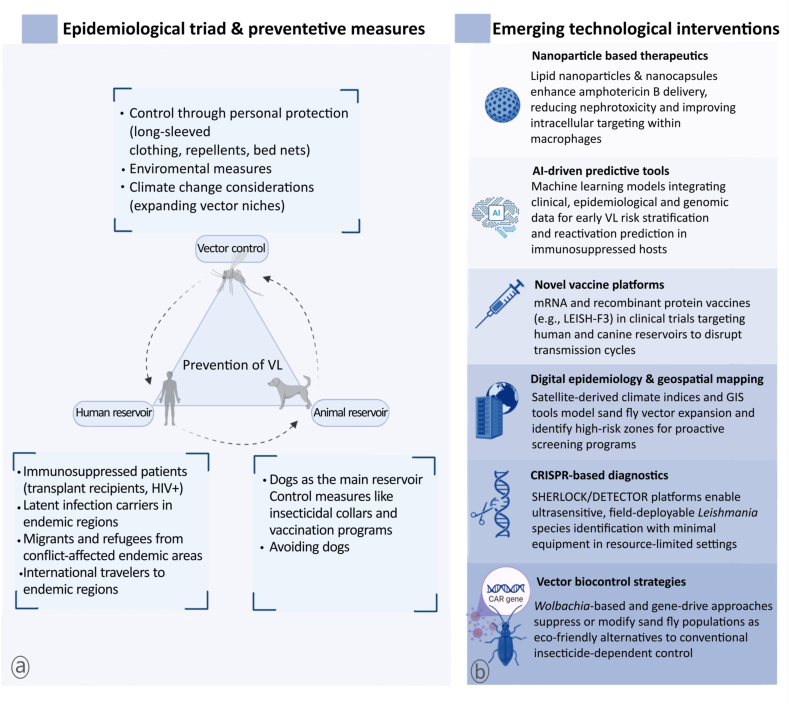
A comprehensive framework showing the interconnected strategies for visceral leishmaniasis control (**a**), spanning host-vector-reservoir dynamics to cutting-edge diagnostic and therapeutic innovations (**b**).

People with latent leishmaniasis infection in endemic regions have contributed to the increase in leishmaniasis cases. In the present situation, there is no clear explanation for the positive PCR results found in the donor and recipient, which makes it difficult to gain a deeper understanding of the disease’s pathogenesis. Research should examine the benefits of screening high-risk recipients and donors in the future to inform informed decisions. In patients at risk, a peripheral blood PCR level for monitoring therapeutic efficacy and secondary prophylaxis could be considered, but further research is needed. The expansion of transplant services in endemic countries and the increase in global mobility will inevitably lead to more leishmaniasis cases among transplant patients, underscoring the need for clinicians to be aware of the risk of this infection affecting their patients (Gajurel et al., 2017; Rocha et al., 2022) (Fig. 7 (a)).

## 12 Prospects and challenges

The management of VL in immunocompromised patients faces profound challenges. From a diagnostic standpoint, standard serological tests such as rK39 often yield false-negative results and are less useful in these individuals, particularly when the CD4 count is very low. This can result in potentially harmful delays in diagnosis (da Silva et al., 2018). Although thrombocytopenic individuals may be candidates for invasive operations like bone marrow aspiration, these procedures are not always possible owing to the potential problems that may arise, such as hemorrhage (Miao et al., 2017). It is of utmost importance to note that the clinical manifestations of the illness can be ambiguous and may be mistaken for other opportunistic diseases (Yadav et al., 2023). In the therapeutic field, the most significant challenge is the very high recurrence rate that occurs even after the original therapy has been effective. This is because the patient’s immune system is unable to completely eliminate the parasite (Tiwari et al., 2024). It might often be difficult to choose the most effective medication regimen. Medicines containing antimony have high toxicities and interact with a wide variety of other medicines. Even if liposomal amphotericin B is a superior choice, there are still concerns over the renal toxicity and the expense of the medication (Saheki et al., 2017). Additionally, there are no established procedures for the duration of treatment or for the use of secondary preventive therapy (prophylaxis) across any of the patient categories. The lack of an effective vaccine is the most significant barrier to preventative efforts. Currently available techniques are limited to vector management and individual protection, both of which are difficult to adhere to consistently (Volpedo et al., 2021).

However, the total number of cases of VL is increasing worldwide due to the lack of an effective vaccine, difficulties in managing a wide spectrum of reservoir hosts and a diverse range of phlebotomine sand fly species, and the development of resistance to the medications of choice (Sharifi et al., 2017). In immunocompromised individuals, the management of VL increasingly relies on sophisticated technologies. This is the future prognosis for the treatment of this condition. Nanotechnology will revolutionize point-of-care diagnostics by enabling ultrasensitive nanosensors. This will reduce the dependence on intrusive procedures, which will be brought about by the revolution. At the same time, nanoparticle-based smart medication delivery systems will enable precise targeting of the parasite, thereby increasing therapy effectiveness and preventing new infections (Tiwari et al., 2023). On the other hand, artificial intelligence will be able to recognize intricate patterns by analyzing large amounts of clinical and epidemiological data. This will enable it to develop prediction tools to identify high-risk patients and make individualized treatment decisions (Fig. 7 (b)) (De Niz et al., 2025). Finally, the confluence of these new technologies will result in protocols that are accurate, economical, and accessible to this vulnerable group.

## 13 Conclusions

Visceral leishmaniasis in immunocompromised patients presents with more aggressive manifestations, a more complex clinical course, and a more limited response to conventional therapies. Current research has shown that an important component of the pathogenesis of VL in this specific group is impaired cellular immunity, particularly a deficiency of T helper cells (CD4+) and a reduced production of essential cytokines such as IFN-γ. This impairment not only impairs the ability to manage the infection but also significantly reduces the sensitivity of traditional serological diagnostic methods (e.g. rK39). As a result, molecular approaches (e.g. PCR) become vital tools for accurate diagnosis. On the other hand, in the therapeutic area, although liposomal amphotericin B continues to maintain its position as first-line therapy in many protocols, its efficacy has been questioned due to the occurrence of frequent disease relapses, the development of drug resistance, and associated toxicities. This is particularly true in patients with profound immunodeficiency. In addition, the lack of effective preventive measures, such as vaccination, prophylactic drugs, and targeted screening programmes, has created a significant gap in the care of these individuals. According to this review’s findings, failure to address the distinctive characteristics of this population not only leads to treatment failure and increased mortality but also, by creating hidden human reservoirs, can play an important role in perpetuating the disease transmission cycle and undermining community-level control programmes. Therefore, the primary focus of future research should be on the development and standardization of rapid, sensitive, and cost-effective molecular diagnostic kits for use at the point of care; the design of clinical trials to evaluate new combination regimens and longer courses of maintenance therapy; and the conduct of epidemiological studies to understand the dynamics of disease transmission and assess the cost-effectiveness of screening programmes in high-risk populations.

## Ethical approval

Not applicable.

## CRediT authorship contribution statement

**Soroush Partovi Moghaddam:** Writing – review & editing, Methodology, Investigation, Formal analysis, Data curation, Validation. **Iraj Sharifi:** Project administration, Conceptualization, Writing - original draft, Writing - review & editing, Formal analysis, Methodology. **Narges Lotfalizadeh:** Conceptualization, Writing - review & editing, Project administration, Methodology, Investigation, Data curation, Funding acquisition. **Shayan Amini:** Writing - review & editing, Methodology, Investigation, Data curation. **Ali Mobaraki:** Writing – review & editing, Formal analysis. **Bita Fazel:** Writing - review & editing, Formal analysis. **Mehdi Bamorovat:** Writing - original draft, Writing - review & editing, Formal analysis, Data curation. **Ahmad Khosravi:** Writing - review & editing, Methodology, Investigation, Data curation. **Baharak Akhtardanesh:** Writing - original draft, Writing - review & editing, Formal analysis, Data curation. **Mehdi Mohebali:** Writing - original draft, Writing - review & editing, Formal analysis, Data curation.

## Statement on the use of AI-assisted technologies

The authors declare that AI-assisted tools were used solely for minor grammatical and language editing to improve the readability of the manuscript. No AI was used for generating scientific content.

## Funding

No funding was received for this study.

## Declaration of competing interests

The authors declare that they have no known competing financial interests or personal relationships that could have appeared to influence the work reported in this paper.

## Data Availability

The data supporting the conclusions of this article are included within the article.
