# Supplementary material for: Revisiting visceral leishmaniasis in immunocompromised patients: Ongoing gaps and advances in diagnostics, therapies, and preventive measures
Source: Curr Res Parasitol Vector Borne Dis. 2026 Apr 9;9:100377. doi: 10.1016/j.crpvbd.2026.100377 (PMC13123496; doi:10.1016/j.crpvbd.2026.100377)
Supplement: Multimedia component 1 [file mmc1.pdf]

Supplementary Table S1. Summarized data for previous studies on the epidemiology of visceral leishmaniasis and HIV co-infection.

Region	Sampling year	Study design	Findings	Reference
North-East Brazil	–	*N* = 134 (35 symptomatic VL-HIV; 75 asymptomatic VL-HIV; and 24 healthy controls)	Higher serum levels of IL-6, IL-10 and IL-17 cytokines were observed in symptomatic co-infected patients compared to asymptomatic and healthy controls. Lymphocytes, CD4 count and hemoglobin levels were also lower in symptomatic individuals, while HIV viral loads were higher.	Guedes et al. (2022)
Brazil	2012–2022	*N* = 1525 confirmed cases of VL	HIV co-infections were detected in 466 (30.55%) patients. Male sex, older age and clinical signs, such as cough, weight loss, were significantly associated with a higher likelihood of a co-infection. On the other hand, symptoms such as splenomegaly, hepatomegaly, edema, fever, jaundice and other co-infections were less frequent among co-infected individuals.	de Souza et al. (2025)
Brazil	2011–2016	*N* = 81 autochthonous cases of VL	10% of patients had VL/HIV co-infection. Lower age was the main difference between the total VL cases and those who were co-infected or died. Clinically, weakness, fever and splenomegaly were more frequent among all VL cases and VL/HIV co-infected individuals. Bacterial infections and bleeding were associated with death due to VL.	Luz et al. (2018)
Brazil	2007–2013	*N* = 126 VL patients	61 (48.4%) of patients were co-infected with HIV/AIDS. Patients with VL with or without HIV/AIDS co-infection were mostly adult men. Diarrhea was more frequent in HIV/AIDS co-infected patients, whereas splenomegaly was more common in patients with VL only. There was a higher rate of VL recurrence and mortality in the HIV/AIDS co-infected patients.	Viana et al. (2017)
Brazil	2007–2010	*N* = 1779 VL patients	33 out of 1779 patients were co-infected with HIV. The incidence of VL/HIV co-infection increased from 0.32/100000 inhabitants in 2007 to 1.08/100000 inhabitants in 2010. There were more male patients in the VL/HIV group than in the VL group. Relapse rate was also considerably higher in the VL/HIV group (9.1%) than in the VL group (1.5%).	Albuquerque et al. (2014)
Brazil	2011–2013	*N* = 90 confirmed VL cases	46 (51%) were HIV co-infected patients. HIV patients had a lower rate of splenomegaly and fever compared with immunocompetent patients. The VL relapse rate in 6 months was 37% among HIV-infected patients, despite receiving secondary prophylaxis. The overall case-fatality rate was 6.6% (4 deaths in the HIV-infected group *vs* 2 deaths in the non-HIV-infected group).	Cota et al. (2014)
Brazil	January 2006-December 2016	*N* = 1171 VL patients	There were 57 cases of VL/HIV co-infection, with emphasis on the year 2013 and the municipality of Santarem, which had the highest number of cases. There was a correlation between co-infection VL/HIV, with significant differences between patients with and without HIV who contracted VL.	Camargo Júnior et al. (2023)

Region	Sampling year	Study design	Findings	Reference
Brazil	–	*N* = 473 VL patients	5.5% of 473 patients were co-infected with HIV. The highest proportion of cases of both VL and VL/HIV was found among men. A higher proportion of VL cases was seen in children aged 0–10 years, whereas co-infection was more common in those aged 18–50 years.	Coutinho et al. (2017)
India	–	*N* = 111 VL patients	A total of 111 patients with VL were screened for HIV infection, and 7 (6.30%) were found to be positive for HIV.	Sinha et al. (2003)
India	–	*N* = 2077 VL patients	In a cohort of 2077 patients aged ≥ 14 years with confirmed VL in Bihar, India, HIV co-infection was identified in 5.6% of cases. Among these, 2.4% represented newly diagnosed HIV infections.	Burza et al. (2014)
India	July 2012-September 2014	*N* = 102 VL patients	57% were found to have HIV infection. Cumulative incidence of all-cause mortality and VL relapse at 6, 12 and 18 months was 11.7%, 14.5%, 16.6%, and 2.5%, 6.0%, 13.9%, respectively. Cumulative incidence of poor outcome at 6, 12, and 18 months was 13.9%,18.4%, and 27.2%, respectively.	Mahajan et al. (2015)
India	2014–2019		Presence of VL-HIV+ and PKDL cases was both associated with a more than 2-fold increase in VL incidence at the village level, with incidence rate ratios of 2.16 and 2.37 for VL-HIV+ and PKDL cases, respectively.	Cloots et al. (2021)
North-West Ethiopia	2010–2015	*N* = 170 VL patients	56% of VL-HIV+ patients were diagnosed with both infections concurrently, while ART coverage at VL diagnosis was 76%, and ART initiation among cured patients reached 67% and 36% in the referral and district hospitals, respectively.	van Griensven et al. (2017)
North-West Ethiopia	January 1999-July 2004	*N* = 212 VL patients	87 (41.0%) were HIV co-infected. Age > 20 years was independently associated with HIV co-infection. The case fatality rates among HIV-positive and HIV-negative kala-azar cases were 39.3% and 13%, respectively.	Mengistu and Ayele (2007)
Iran	–	*N* = 49 HIV/AIDS patients; Diagnostic method: HIV: ELISA, Western blot; VL: DAT	9 (18.4%) patients were seropositive according to DAT. The most predominant signs and symptoms of co-infection of VL in HIV-positive patients were pneumonia (*n* = 2), hepatosplenomegaly (*n* = 2), lymphadenopathy (*n* = 2), anemia (*n* = 1), prolonged fever (*n* = 1) and cachexia (*n* = 1).	Shafiei et al. (2014)
Iran	2017	*N* = 251 HIV/AIDS patients; Diagnostic method: VL: ELISA, PCR	19 out of 251 (7.6%) HIV-infected patients were found to be infected with *Leishmania*, using serological or molecular tests. The CD4+ counts of 17 out of 19 HIV/*Leishmania* co-infected patients were normal, whereas the CD4+ T	Rezaei et al. (2018)

Region	Sampling year	Study design	Findings	Reference
			cell counts of two seropositive cases, which were also PCR-positive, were low (< 200 cells/μl).	
Morocco	April 2016-November 2016	*N* = 200 HIV/AIDS patients; Diagnostic method: VL: DME, culture, IFI, PCR	Circulating anti-*Leishmania* antibodies were detected by IFI serology in 10 of the 200 patients infected with HIV, presenting a 5% rate of *L. infantum* infection in HIV-infected patients. The amastigote form of the parasite was observed in 3% (6/200) of the HIV patients after DME of the white blood cell layer under an optical microscope. The promastigote form of the parasite was observed under the light microscope in 2.5% (5/200) of the HIV patients by direct examination of the culture.	Echchakery et al. (2018)

Abbreviations: VL, visceral leishmaniasis; HIV, human immunodeficiency virus; IL, interleukin; PCR, polymerase chain reaction; DME, direct microscopic examination; IFI, indirect immunofluorescence; ELISA, enzyme-linked immunosorbent assay; CD4, cluster of differentiation 4; ART, antiretroviral treatment; DAT, direct agglutination test.

References

Albuquerque, L. C.P.D., Mendonça, I.R., Cardoso, P.N., Baldaçara, L.R., Borges, M.R.M.M., Borges, J.D.C., Pranchevicius, M.C.D.S., 2014. HIV/AIDS-related visceral leishmaniasis: a clinical and epidemiological description of visceral leishmaniasis in northern Brazil. Rev. Soc. Bras. Med. Trop. 47, 38-46.

Burza, S., Mahajan, R., Sanz, M.G., Sunyoto, T., Kumar, R., Mitra, G., Lima, M.A., 2014. HIV and visceral leishmaniasis coinfection in Bihar, India: an underrecognized and underdiagnosed threat against elimination. Clin. Infect. Dis. 59, 552-555.

Camargo Júnior, R.N.C., Sarmento Gomes, J.S., Corrêa Carvalho, M.C., Chalkidis, H.D.M., Silva, W.C.D., et al., 2023. Visceral leishmaniasis associated with HIV coinfection in Pará, Brazil. HIV AIDS (Aukl.) 15, 247-255.

Cloots, K., Marino, P., Burza, S., Gill, N., Boelaert, M., Hasker, E., 2021. Visceral leishmaniasis-HIV coinfection as a predictor of increased *Leishmania* transmission at the village level in Bihar, India. Front. Cell. Infect. Microbiol. 11, 604117.

Cota, G.F., de Sousa, M.R., de Mendonça, A.L.P., Patrocinio, A., Assunção, L.S., de Faria, S.R., Rabello, A., 2014. *Leishmania*-HIV co-infection: clinical presentation and outcomes in an urban area in Brazil. PLoS Negl. Trop. Dis. 8, e2816.

Coutinho, J.V.S.C., Santos, F.S.D., Ribeiro, R.D.S.P., Oliveira, I.B.B., Dantas, V.B., Santos, A.B.F.S., Tauhata, J.R., 2017. Visceral leishmaniasis and leishmaniasis-HIV coinfection: comparative study. Rev. Soc. Bras. Med. Trop. 50, 670-674.

de Souza, R.M., Tuon, F.F., Dantas, L.R., Imamura, R., Funari, A.P., Celeste, B.J., Amato, V.S., 2025. Reactivation of cutaneous leishmaniasis in the context of non-HIV immunosuppression: a literature analysis. Trans. R. Soc. Trop. Med. Hyg. 119, 922-925.

Echchakery, M., Nieto, J., Boussaa, S., El Fajali, N., Ortega, S., Souhail, K., et al., 2018. Asymptomatic carriers of Leishmania infantum in patients infected with human immunodeficiency virus (HIV) in Morocco. Parasitol. Res. 117, 1237-1244.

Guedes, D.L., Silva, E.D.D., Castro, M.C.A.B., Júnior, W.L.B., Ibarra-Meneses, A.V., Tsoumanis, A., et al., 2022. Comparison of serum cytokine levels in symptomatic and asymptomatic HIV-*Leishmania* coinfected individuals from a Brazilian visceral leishmaniasis endemic area. PLoS Negl. Trop. Dis. 16, e0010542.

Luz, J.G.G., Naves, D.B., Carvalho, A.G.D., Meira, G.A., Dias, J.V.L., Fontes, C.J.F., 2018. Visceral leishmaniasis in a Brazilian endemic area: an overview of occurrence, HIV coinfection and lethality. Rev. Inst. Med. Trop. Sao Paulo. 60, e12.

Mahajan, R., Das, P., Isaakidis, P., Sunyoto, T., Sagili, K.D., Lima, M.A., et al., 2015. Combination treatment for visceral leishmaniasis patients coinfected with human immunodeficiency virus in India. Clin. Infect. Dis. 61, 1255-1262.

Mengistu, G., Ayele, B., 2007. Visceral leishmaniasis and HIV co-infection in patients admitted to Gondar University Hospital, northwest Ethiopia. Ethiop. J. Health Dev. 21, 1-108.

Rezaei, Z., Sarkari, B., Dehghani, M., Layegh Gigloo, A., Afrashteh, M., 2018. High frequency of subclinical *Leishmania* infection among HIV-infected patients living in the endemic areas of visceral leishmaniasis in Fars Province, southern Iran. Parasitol. Res. 117, 2591-2595.

Shafiei, R., Mohebali, M., Akhoundi, B., Galian, M.S., Kalantar, F., Ashkan, S., et al., 2014. Emergence of co-infection of visceral leishmaniasis in HIV-positive patients in northeast Iran: a preliminary study. Travel Med. Infect. Dis. 12, 173-178.

Sinha, P.K., Rabidas, V.N., Pandey, K., Verma, N., Gupta, A.K., Ranjan, A., et al., 2003. Visceral leishmaniasis and HIV coinfection in Bihar, India. J. Acquir. Immune Defic. Syndr. 32, 115-116.

van Griensven, J., Simegn, T., Endris, M., Diro, E., 2017. Visceral leishmaniasis and HIV co-infection in Northwest Ethiopia: antiretroviral treatment and burden of disease among patients enrolled in HIV care. Am. J. Trop. Med. Hyg. 98, 486.

Viana, G.M.D.C., Silva, M.A.C.N.D., Garcia, J.V.D.S., Guimarães, H.D., Arcos, G.F., Santos, A.V.A., et al., 2017. Epidemiological profile of patients co-infected with visceral leishmaniasis and HIV/AIDS in Northeast, Brazil. Rev. Soc. Bras. Med. Trop. 50, 613-620.
